# Implementation of a Systematic Accountability Framework in 2014 to Improve the Performance of the Nigerian Polio Program

**DOI:** 10.1093/infdis/jiv492

**Published:** 2016-04-02

**Authors:** Sisay G. Tegegne, Pascal MKanda, Yared G. Yehualashet, Tesfaye B. Erbeto, Kebba Touray, Peter Nsubuga, Richard Banda, Rui G. Vaz

**Affiliations:** 1 World Health Organization, Country Representative Office, Abuja, Nigeria; 2 World Health Organization, Regional Office for Africa, Brazzaville, Congo; 3 Global Public Health Solutions, Atlanta, Georgia

**Keywords:** accountability framework, selected deliverables, administrative actions, staff performance, program performance

## Abstract

***Background.*** An accountability framework is a central feature of managing human and financial resources. One of its primary goals is to improve program performance through close monitoring of selected priority activities. The principal objective of this study was to determine the contribution of a systematic accountability framework to improving the performance of the World Health Organization (WHO)–Nigeria polio program staff, as well as the program itself.

***Methods.*** The effect of implementation of the accountability framework was evaluated using data on administrative actions and select process indicators associated with acute flaccid paralysis (AFP) surveillance, routine immunization, and polio supplemental immunization activities. Data were collected in 2014 during supportive supervision, using Magpi software (a company that provides service to collect data using mobile phones). A total of 2500 staff were studied.

***Results.*** Data on administrative actions and process indicators from quarters 2–4 in 2014 were compared. With respect to administrative actions, 1631 personnel (74%) received positive feedback (written or verbal commendation) in quarter 4 through the accountability framework, compared with 1569 (73%) and 1152 (61%) during quarters 3 and 2, respectively. These findings accorded with data on process indicators associated with AFP surveillance and routine immunization, showing statistically significant improvements in staff performance at the end of quarter 4, compared with other quarters.

***Conclusions.*** Improvements in staff performance and process indicators were observed for the WHO-Nigeria polio program after implementation of a systematic accountability framework.

In 2014, the World Health Organization (WHO) declared that polio is a public health emergency of international concern, as defined by the WHO's international health regulations [2]. Accordingly in addition to the four proven strategies namely strong routine immunization, supplemental immunization activity, mop up activities and strong surveillance other new strategies and the implementation of accountability mechanism are crucial [1, 2].

The WHO follows a results-based management approach that calls for delegated responsibility, authority, and accountability in a decentralized environment at all levels of the organization. This means that decisions on the use of financial and other resources are taken by managers at all levels in all locations. Therefore, accountability is at the core of performance evaluation. In addition, tracking health progress at the global and country level through adequate and quality data has been given attention at all levels to foster accountability [3, 4].

In addition to these guiding principles and policies, studies were conducted to determine the link between accountability principles and the results of their implementation. Work by Cleary et al suggests that, while resources and capacity are necessary conditions of functionality, the link between organizational culture, relationships, and accountability processes is a key consideration in any intervention and context [5].

One focus of accountability is improving program performance through close monitoring of selected priority activities. Research conducted in Tanzania showed that among other things setting priorities requires accountability and transparency in the system [6, 7].

Accountability should also focus on managing human resources. A study of the impact of targeted programs on health systems, using polio as a case study, revealed a global gap in the documentation of the basic approaches in managing human resources [8].

Another arm of accountability addresses how financial resources are managed and used for program performance. A report on the financial resource requirement for the global polio eradication initiative 2013–2018 stated that $250 million per year will be required by Nigeria for successful completion of the milestones in the polio endgame strategy. This huge resource requirement has put a responsibility on donor recipients to demonstrate results for the investment in the program. A review of the transparency and accountability initiative by Mulley on donor aid identified that the overall principle that governs donor aid is transparency and accountability for commitments and results [9, 10].

Many studies have been conducted to determine the reasons for the challenges associated with polio eradication in Nigeria. Tagbo cited a poor accountability framework as one of the major challenges in achieving polio eradication in Nigeria [11]. The Independent Monitoring Board of the Global Polio Eradication Initiative, in October 2014, and the 27th Expert Review Committee on Polio Eradication and Routine Immunization in Nigeria reports and recommendations also elaborated the need for systematic accountability in Nigeria, in which people are held responsible for delivering results [12, 13]. The 2014 Nigeria National Polio Emergency Plan emphasized strict accountability at all levels as one of the strategic priorities.

The WHO, as the technical organization and implementing partner, is responsible for monitoring activities, through systematic implementation of an accountability framework, this is due to emphasis in the emergency plan of action for Polio for the country [14]. As one of the major stakeholders in the polio eradication initiative in Nigeria, the WHO country office manages >2500 staff throughout the country for polio and other health programs. But how do these personnel function in improving program performance, and how does the implementation of systematic accountability contribute to improved individual staff performance? We set out to determine the influence of systematic accountability framework implementation on the performance of WHO-Nigeria staff and the polio program.

## METHODS

An accountability framework was implemented in the Nigeria polio program by the WHO Nigeria Country Office in 2014. We used select indicators on acute flaccid paralysis (AFP) surveillance, routine immunization, and polio supplemental immunization activities (SIAs) to measure the impact of WHO officer support on the performance of the polio eradication initiative in Nigeria and the impact of accountability framework implementation on the performance of individual WHO officers over 1 year.

### Description of the Accountability Framework

**Figure 1.**
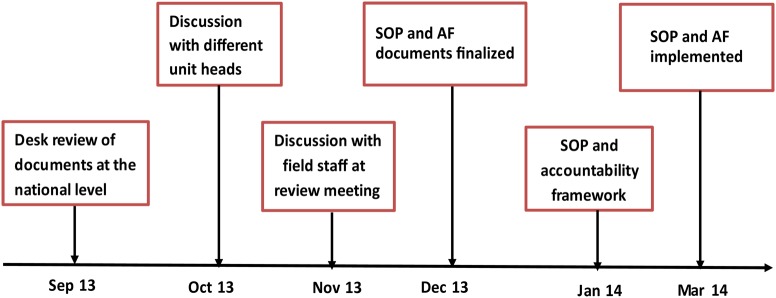
Milestones of accountability framework (AF) implementation. Abbreviation: SOP, standard operating procedures.

A systematic monitoring and evaluation system that measured staff performance by collecting relevant evidence and tracking framework implementation was discussed in detail by the management team in the country office during the second quarter of 2013 (Figure 1). A desk review of documents at the national level was conducted in September 2013, and a checklist was developed to guide discussion of a draft of standard operating procedures with unit heads of surveillance, routine immunization, and SIAs in October 2013. During November and December 2013, the discussion was expanded to include all staff in the immunization vaccines and emergency cluster of WHO-Nigeria. Indicators that cut across different program areas were selected to serve as deliverables by which program performance and staff performance would be measured. In January 2014, implementation of the accountability framework began. The framework produced monthly and quarterly feedback by means of different administrative actions, depending on staff performance.

### Study Population

All WHO officers at the field level, namely field volunteers, local government area (LGA) facilitators, cluster coordinators (ie, medical officers who monitor and supervise ≥2 districts that make a cluster), and state coordinators, were included. A total of 2500 staff were studied.

### Data Collection

Data were collected via Magpi's mobile data collection platform, which uses mobile phones and a real-time cloud-based system with global positioning system (GPS) coordinates and time stamping [15]. A total of 52 variables were used to collect data on surveillance and immunization activities at the health facilities visited and provide feedback. The WHO staff have used this mobile-based data collection tool to collect data regularly from health facilities throughout the country. Changes over time in select process indicators were recorded during supervision visits by WHO officers at health facilities throughout the country . Data from January 2014 to December 2014 were used to determine the impact of accountability framework implementation on program performance. Data validation involving data collected during randomly selected supervision visits was also done through the existing monitoring and evaluation system in the country.

To measure WHO staff performance over time, the accountability framework encompassed selected key performance indicators, which were a mix of process and outcome indicators. The indicators were extracted from surveillance activities, routine immunization activities, and polio SIAs. The accountability framework used data collected using Magpi, data from the AFP surveillance database, and data from the polio SIA database. The tool developed for the accountability framework triangulates these data sets and produces results that measure individual staff performance against the selected indicators. We developed and used the following color-coded framework to guide management decisions: >3 red marks in the list of indicators resulted in termination of the contract; 2 red marks, despite monthly feedback to improve performance, resulted in written warning; ≥2 yellow marks resulted in a verbal warning or a recommendation to improve performance; and green marks resulted in an appreciation letter or verbal commendations from the management. Actions taken every quarter were monitored regularly (Table 1).

**Table 1.** Target Thresholds for Indicators in the Accountability Framework Implemented in the Nigeria Polio Program, 2014

Indicator	Threshold	Rating or Observation	
		From Officer 1	From Officer 2
Quarterly polio risk assessments submitted in timely fashion, %	≥80	100	100
Cases detected by active case searching, % of expected value	90	93	89
60-d follow-up reports submitted in timely fashion, %^a^	≥90	…	…
AFP cases reported, no.	…	7	3
AFP cases verified, % of reported cases	≥90	78	0
AFP cases verified within 7 d, % of reported cases	≥80	100	60
TC of data quality self-assessment, % of LGA	≥80	100	90
LGAs supervised, no.	…	2	2
Wards with updated REW strategy, %	80	ND	ND
Fixed sessions conducted, % of planned sessions	80	71	36
Outreach sessions conducted, % of planned sessions	80	67	38
Sessions monitored, % of planned sessions	80	75	100
HFs visited, no.	…	35	34
HFs with vaccine stock out, %	<10	19	9
HFs with updated monitoring chart, %	≥80	59	51
Wards, no.	…	22	22
Microplans validated, %	≥80	100	100
Validated teams for composition, %	≥80	100	100
LGAs with ≥80% LQAS coverage, %	≥80	100	100
Demand creation data submitted in timely fashion, %	100	100	100
MDD records submitted, %	90	130	126
Activity reports submitted in timely fashion, %	100	100	67

Abbreviations: AFP, acute flaccid paralysis; HF, health facility; LGA, local government authority; LQAS, lot quality-assurance sampling; MDD, mobile device data; ND, not determined; REW, Reaching Every District; TC, timeliness and completeness.

^a^ Defined as receipt of the report 61–70 d after the start of the follow-up period.

The databases used as inputs for the accountability framework monitoring tool were as follows: case-based surveillance for AFP, case verification of reported AFP cases, the SIA monitoring dashboard, and real-time data generated from supportive supervision visits, using mobile devices. The data were collected and analyzed and feedback given monthly to individual WHO officers. The same tool was used to analyze performance of officers on quarterly basis for administrative action for accountability.

We used the following variables in our analyses: administrative actions taken (appreciation letter, verbal commendation, discussion to improve, verbal warning, written warning, and contract nonrenewal), health worker knowledge of AFP case definition, performance of supportive supervision or active case searching for AFP, availability of immunization monitoring chart in health facilities, and performance of data quality self-assessment in visited facilities.

### Data Analysis

To measure WHO staff performance over time, the administrative actions taken on individual staff members and the conduct of 2 major activities—active case searching and data quality self-assessment at health facilities—were compared. While we took the knowledge of AFP case definition by health workers in the health facilities and availability of updated monitoring chart in health facilities to compare the impact of implementation of systematic accountability framework on program performance. Paired *t* tests were performed to evaluate the statistical significance of differences in program performance between quarter 2 (April to June of 2014) and quarter 4 (October to December of 2014). This data analysis was done on the premises of direct relation on changes occurring in this critical surveillance and routine immunization indicators with a corresponding change in program performance.

To measure the impact of the accountability framework on individual WHO staff performance, we compared the changes in different administrative actions taken over time. We also compared the changes in officer performance over time, using active case searching for AFP cases and data quality self assessment at health facilities.

## RESULTS

The accountability framework was implemented in phases, yielding a steady increase in the number of enrolled staff per quarter: 2215 personnel enrolled in the system and received feedback in quarter 4, compared with 549 in quarter 1. A total of 1631 personnel (74%) received positive feedback (appreciation letter and verbal commendation) in quarter 4, compared with 1569 (73%) and 1152 (61%) in quarters 3 and 2, respectively. During quarter 4, 203 personnel (9%) received a verbal and written warning to improve performance, while 117 (5%) and 302 (16%) received such feedback in quarters 3 and 2, respectively. On the basis of management decisions, the contracts for 300 staff members were not renewed, owing to persistent underperformance despite receipt of feedback to improve (Table 2).

**Table 2.** Administrative Actions Taken on the Basis of the Performance of Nigeria Polio Program Staff During 2014, by Quarter

Administrative Action	Quarter 1, Staff, No (%) (n = 549)	Quarter 2, Staff, No (%) (n = 1878)	Quarter 3, Staff, No (%) (n = 2137)	Quarter 4, Staff, No (%) (n = 2215)
Written/verbal commendation	248 (45)	1152 (61)	1569 (73)	1631 (74)
Discussion to improve	57 (10)	222 (12)	439 (21)	369 (17)
Written/verbal warning	170 (31)	302 (16)	117 (5)	203 (9)
Contract nonrenewal	74 (13)	202 (11)	12 (1)	12 (1)

**Figure 2.**
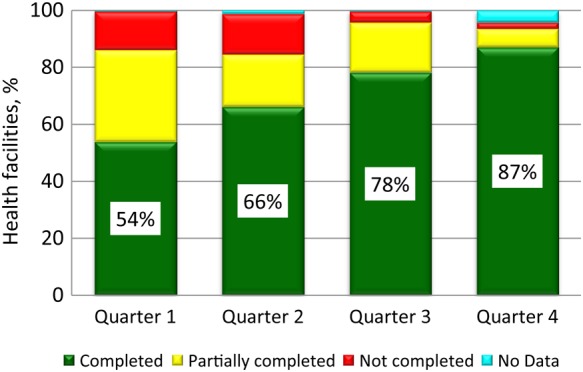
Percentage of the targeted number of Nigerian health facilities supervised by World Health Organization (WHO) staff at which active case searching was completed, partially completed, or not completed in 2014, by quarter.

In quarter 4, active case searching for AFP at health facilities supervised by WHO officers was completed in accordance with standard operative procedures at 87% of health facilities, compared with 66% in quarter 2 (Figure 2). For verification of supervision visits, all visits were tracked using GPS technology.

**Figure 3.**
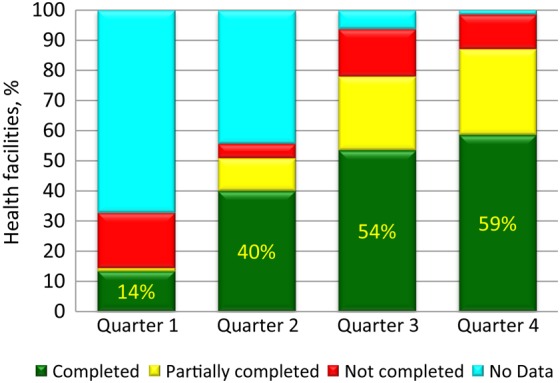
Percentage of Nigerian health facilities supervised by World Health Organization (WHO) staff in which data quality self-assessments were completed, partially completed, or not completed in 2014, by quarter.

Routine immunization performance was measured by data quality self-assessments conducted by WHO officers during supervision visits to health facilities. The cumulative percentage of facilities in which data quality self-assessments were completed improved from 40% in quarter 2 to 59% in quarter 4 (Figure 3). The availability of updated immunization monitoring charts improved over time among facilities where supervision visits occurred, from 62% during January–June to 86% in December 2014 (Table 3) and from a mean (±SD) of 68% ± 0.21% during quarter 2 to 83% ± 0.13% during quarter 4 (*P* = .001). The mean proportion (±SD) of health workers who knew the AFP case definition was 82% ± 0.25% in quarter 2, compared with 96% ± 0.05% in quarter 4 (*P* = .002).

**Table 3.** Availability of Immunization Monitoring Charts in Nigerian Health Facilities During Supervision Visits by World Health Organization Officers in 2014, by Month

Month(s)	Facilities With Updated Monitoring Charts, %
January–June (baseline)	62
July	71
August	71
September	77
October	78
November	80
December	86

## DISCUSSION

We sought to determine the contribution of the accountability framework to improving staff performance in the Nigeria polio program. We found that staff performance during 2014 improved at the end of quarter 4, compared with other quarters, as measured by the number of supervision visits to priority health facilities and the availability of updated immunization monitoring charts at the facilities. We also found that the accountability framework contributed to program implementation, as shown by a statistically significant change in the process indicators of both AFP surveillance and routine immunization.

Ours is not the first study on accountability process and its influence. An earlier review by Brett et al on participation and accountability showed similar results of an accountability framework implemented to foster transparency and improve performance [16]. Our approach is different because it evaluated changes in health program outcomes, as well as individual staff performance.

The increase in the proportion of staff who achieved all indicators revealed the importance of feedback during implementation of the accountability. The feedback communicated through in-depth discussion of each performance indicator with each individual helped to develop confidence among the staff and management. Individual staff received regular feedback every quarter on their performance, and training and higher-level supervision was guided based on these results.

The primary limitation to the generalization of our findings is that the accountability framework was not implemented in all Nigerian states during the first quarter of 2014. Therefore, we analyzed data on staff performance and program improvement beginning with quarter 2, when >84% of field staff members were enrolled in the framework. In addition, accessibility of health facilities in security-compromised areas made it difficult to evaluate the impact of accountability among polio programs in these areas. Finally, the accountability framework approach must go beyond the polio program, with the goal of enhancing staff performance and program implementation across all immunization programs.

Furthermore, in any system of accountability, openness and transparency are pillars. A study by Lawrence and Nezhad revealed that increased transparency can encourage greater donations by assuring donors that their donations are reaching the desired populations and increase overall quality service [17]. To ensure good practices in staff management, we recommend a qualitative study of the influence of transparency on the staff monitored by an accountability framework.

In conclusion, the systematic implementation of an accountability framework in Nigeria and its influence on improving program performance is one of the best practices in polio legacy activities. As Nigeria gets closer toward interrupting transmission of polio, there is a dire need to maintain and improve the accountability framework to ensure successful achievement of polio eradication initiative objectives.
